# Periodic albinism of a widely used albino mutant of *Xenopus laevis* caused by deletion of two exons in the Hermansky–Pudlak syndrome type 4 gene

**DOI:** 10.1111/gtc.12818

**Published:** 2020-11-28

**Authors:** Toshihiko Fukuzawa

**Affiliations:** ^1^ Department of Biology Keio University Yokohama Japan

**Keywords:** albinism, Hermansky–Pudlak syndrome, melanophores, mutation, periodic albino, retinal pigment epithelium, *Xenopus laevis*

## Abstract

The periodic albino mutant of *Xenopus laevis* is a recessive mutant, in which reduced amounts of melanin appear in the retinal pigment epithelium (RPE) and in melanophores at the late embryonic stage, after which both RPE and melanophores gradually depigment. Three types of pigment cells (melanophores, iridophores and xanthophores) have been reported to be affected in this albino. However, the causative gene of the periodic albinism remains unknown. Hermansky–Pudlak syndrome (HPS) is an autosomal recessive disorder that affects humans and mice, which is caused by defective biogenesis of lysosome‐related organelles (LROs). Two subgenomes (L and S) are present in the allotetraploid frog *X. laevis*. Comparison of genes between the chromosomes 1L and 1S revealed that the HPS type 4 (*hps4*) gene was present only in chromosome 1L. In the albino mutant, a 1.9 kb genomic deletion in the *hps4.L* gene including exons 7 and 8 caused a premature stop codon to create a truncated Hps4 protein. Injection of wild‐type *hps4.L* mRNA into mutant embryos rescued the albino phenotype. These findings indicate that *hps4* is a causative gene for the periodic albinism in *X. laevis*. The phenotype of this mutant should be reassessed from the perspective of LRO biogenesis.

## Graphical abstract

Periodic albino mutant of *Xenopus laevis* has been widely used for experiments in biology; however, the causative gene of this mutant remains unknown. This study identifies *hps4* gene (Hermansky–Pudlak syndrome type 4), as being responsible for the albino mutant of *X. laevis*. This mutant may be useful for studying the component of the biogenesis of lysosome‐related organelles, which are encoded by *hps4* gene.

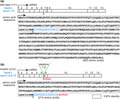

## 1 INTRODUCTION

The periodic albino mutant (*a^p^/a^p^*) of *Xenopus laevis* was reported to appear naturally in Moscow in 1972 (Hoperskaya, 1975). This albino strain can be obtained commercially, and has been widely used for experiments such as reciprocal grafting and in situ hybridization, because eggs and embryos of this mutant do not contain melanin and are easily distinguished from wild‐type cells. In the periodic albino mutant, reduced amounts of melanin appear in the retinal pigment epithelium (RPE) and melanophores at the late embryonic stage, after which both RPE and melanophores gradually depigment during metamorphosis (Hoperskaya, 1975, 1981). Differentiation and pigment organelle formation of three types of pigment cells (melanophores, iridophores and xanthophores) have been reported to be affected in the periodic albino mutant (Fukuzawa, 2006; Fukuzawa & Ide, 1986; Hoperskaya, 1981; MacMillan, 1979; MacMillan & Gordon, 1981; Seldenrijk et al., 1982). In addition, white pigment cells that arise from melanophore precursors, and accumulate reflecting platelets characteristic of iridophores, specifically appear in the periodic albino and are localized where melanophores would normally differentiate in the wild type (Fukuzawa, 2004, 2010, 2015). Crossing experiments show that the periodic albino is a recessive mutant (Hoperskaya, 1975); however, the causative gene is still unknown.

Hermansky–Pudlak syndrome (HPS) is an autosomal recessive disorder affecting humans and mice and demonstrates reduced pigmentation of the eyes and skin (Nguyen et al., 2002; Suzuki et al., 2002). The *HPS4* gene encodes a component of the biogenesis of lysosome‐related organelles (LROs) such as melanosomes and platelet‐dense granules (Carmona‐Rivera et al., 2013; Gerondopoulos et al., 2012; Martina et al., 2003; Nazarian et al., 2003; Wei & Li, 2012). It is known that *HPS* mutations cause defects in both melanosomal and nonmelanosomal LROs in mammals (Wei & Li, 2012).

In the allotetraploid frog *X. laevis*, a long (L) and short (S) subgenome (chromosomes) is present (Matsuda et al., 2015; Session et al., 2016). It has been shown that homoeologous genes are present in both L and S subgenome chromosomes in *X. laevis*, although certain genes are lost in the chromosomes of subgenome S (Session et al., 2016). Comparison of the genome sequences between chromosomes 1L and 1S showed that the *hps4* gene was present only in chromosome 1L. The *hps4.L* gene in the wild‐type *X. laevis* consists of 13 exons interrupted by 12 introns. Since the *hps4.L* mRNA of the periodic albino mutant was found to lack exons 7 and 8, genomic sequencing of the *hps4.L* gene between exons 6 and 9 was carried out. DNA sequencing revealed that a 1.9 kb deletion in the *hps4.L* gene including exons 7 and 8 caused a premature stop codon to create a truncated Hps4 protein in the periodic albino.

Rescue experiments were carried out to prove that the *hps4.L* gene is responsible for this mutation. Injection of wild‐type *hps4.L* mRNA, but not mutant *hps4.L* mRNA, into mutant embryos, rescued the albino phenotype.

These findings show that *hps4* is a causative gene for the periodic albino mutant of *X. laevis*. To my knowledge, this is the first report of *hps* mutation in the allotetraploid frog *X. laevis*. The possible effects of Hps4 dysfunction on pigment organellogenesis in the periodic albino are discussed.

## 2 RESULTS AND DISCUSSION

### 2.1 Reduced amounts of melanin appear at later stages in mutant embryos when compared with wild‐type embryos

**FIGURE 1:**
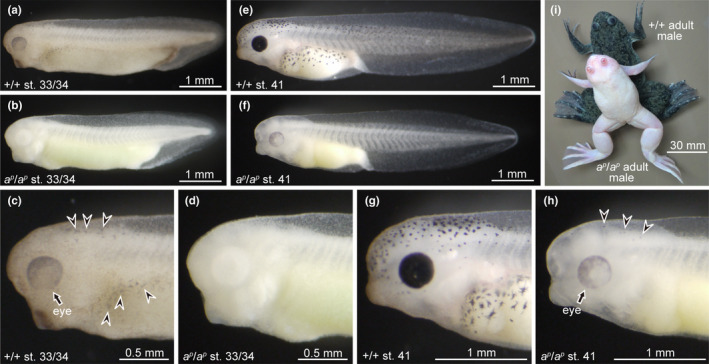
The time of melanin appearance in the eyes and melanophores in the periodic albino mutant is later than that of the wild type. (a–d) Stage 33/34 embryos; (e–h) stage 41 embryos; (i) adult frogs. Photographs of (c), (d), (g) and (h) are enlarged images of the head and trunk regions in (a), (b), (e) and (f), respectively. (a, c, e, g, i) Wild‐type *Xenopus laevis*; (b, d, f, h, i) mutant *X. laevis*. Melanin appears in the eyes (c, arrow) and melanophores (c, arrowheads) at stage 33/34 in the wild type (a, c), but not in the mutant (b, d). Small amounts of melanin begin to appear in the eyes (h, arrow) and melanophores (h, arrowheads) at stage 41 in the mutant (f, h). Although melanin‐containing cells do not disappear in the wild type even after metamorphosis, they almost disappear during metamorphosis in the mutant (i)

Embryos and adults of wild‐type (+/+) and periodic albino mutant (*a^p^/a^p^*) *X. laevis* are shown in Figure 1. In the wild‐type phenotype, dendritic melanophores appear in the head and trunk regions at stage 33/34 (Figure 1a,c). Melanin expression is also evident in the eyes of the wild type at this stage (Figure 1c). In contrast, melanin does not appear in mutant embryos at stage 33/34 (Figure 1b,d). Melanin is observed in the eyes, and melanophores of mutant embryos at stage 41 (Figure 1h), whereas both the eyes and melanophores in wild‐type embryos become heavily pigmented by this stage (Figure 1e,g). Although wild‐type melanophores are darkly pigmented and are mainly dendritic (Figure 1g), mutant melanophores are punctate and pale in color (Figure 1h) at stage 41. Previous reports have shown that melanophores of the periodic albino contain many premelanosomes and immature melanosomes, instead of fully melanized melanosomes (Fukuzawa, 2015; Fukuzawa & Ide, 1986; Seldenrijk et al., 1982).

Depigmentation in RPE and melanophores occurs during metamorphosis in the mutant, but not in the wild type (Hoperskaya, 1975, 1981). Therefore, adult periodic albino frogs have little or no melanin in the eyes and skin (Figure 1i). It has also been reported that melanosomes and premelanosomes are absent in eggs of periodic albino mutant *X. laevis* (Bluemink & Hoperskaya, 1975).

### 2.2 The *hps4* gene is present only in chromosome 1L in *Xenopus laevis*

**FIGURE 2:**
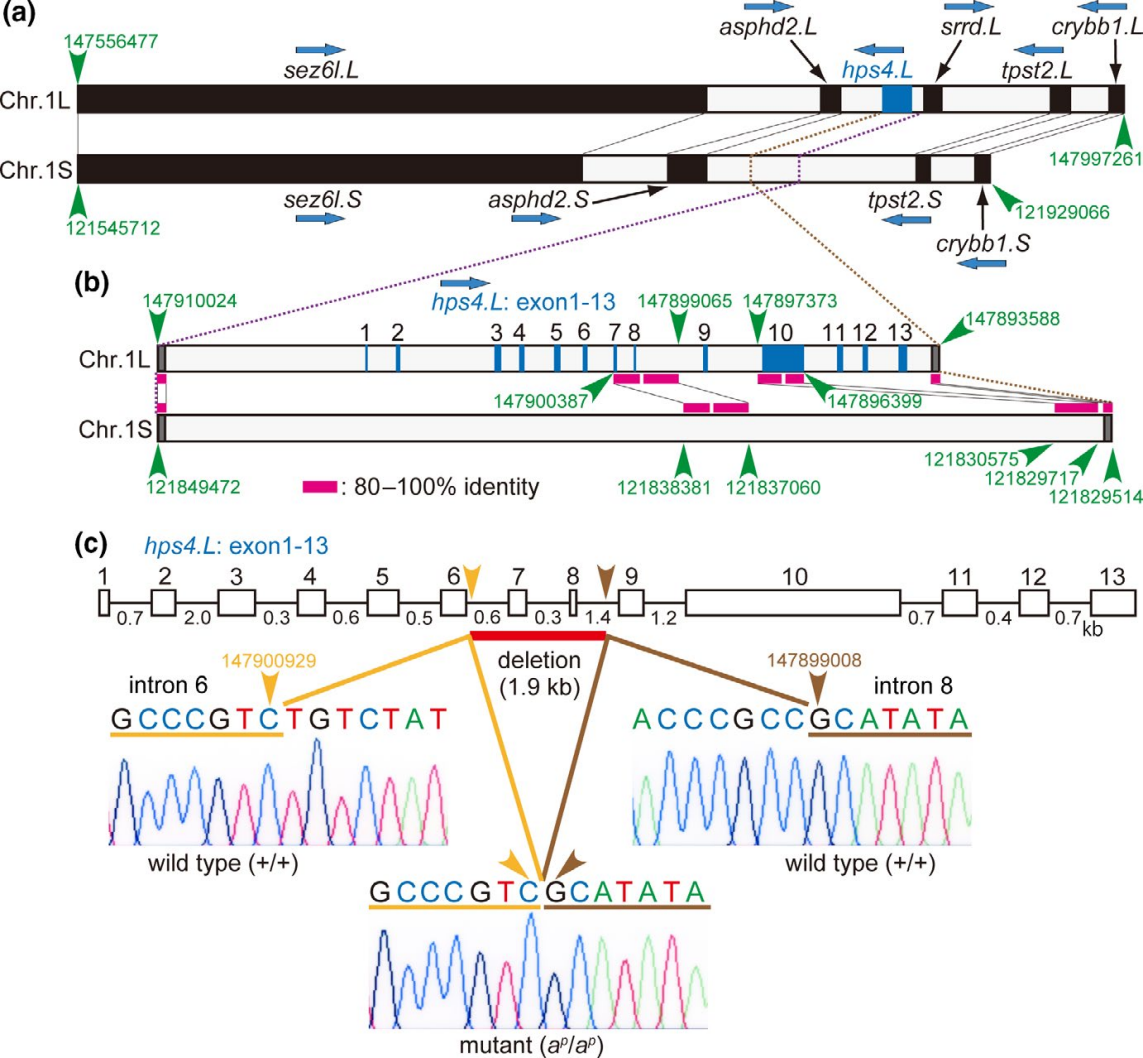
The *hps4* gene is present only in chromosome 1L, and a 1.9 kb genomic deletion including two exons in the *hps4.L* gene occurs in the periodic albino mutant. (a) Schematic representation of *Xenopus laevis* genes in chromosome 1L containing *hps4* locus and in chromosome 1S (v9.2, Xenbase). (b) Alignment of DNA sequences between *hps4.L* region in chromosome 1L and the corresponding position in chromosome 1S. (c) Schematic representation of *hps4.L* gene in the wild type and in the mutant. Wild‐type *hps4.L* gene consists of 13 exons interrupted by 12 introns. DNA chromatograms show a 1.9 kb deletion between introns 6 and 8 in the *hps4.L* gene in the mutant (c, arrowheads). Accession numbers: +/+ *hps4.L* gene between exons 6 and 9, LC577764 (DDBJ); *a^p^/a^p^ hps4.L* gene between exons 6 and 9, LC577765 (DDBJ)

In *X. laevis*, homoeologous genes are present in both L and S subgenome chromosomes, although certain genes are lost in the chromosomes of the S subgenome, which are shorter in length than chromosomes of the L subgenome (Session et al., 2016). Homoeologous genes such as *sez6l*, *asphd2*, *tpst2* and *crybb1* are present in both chromosomes 1L and 1S in the same order (Figure 2a). The *hps4.L* gene consisting of 13 exons (Figure 2b) is located between *asphd2.L* and *srrd.L* in chromosome 1L (Figure 2a). To check whether the *hps4* gene is present in chromosome 1S, a sequence alignment was carried out between the *hps4.L* region in chromosome 1L and the corresponding position in chromosome 1S (Figure 2b). Most of the exons in the *hps4.L* gene were absent in chromosome 1S, although some homoeologous sequences of the *hps4.L* gene (exons 7, 8 and 10) were present in chromosome 1S (Figure 2b). Using Xenbase blast (*X. laevis* genome v9.2), the *hps4* gene was found only in chromosome 1L. These data show that the *hps4* gene is present only in chromosome 1L in *X. laevis*.

### 2.3 Deletion of two exons in the *hps4.L* gene causes a premature stop codon to create a truncated Hps4 protein in the periodic albino

**FIGURE 3:**
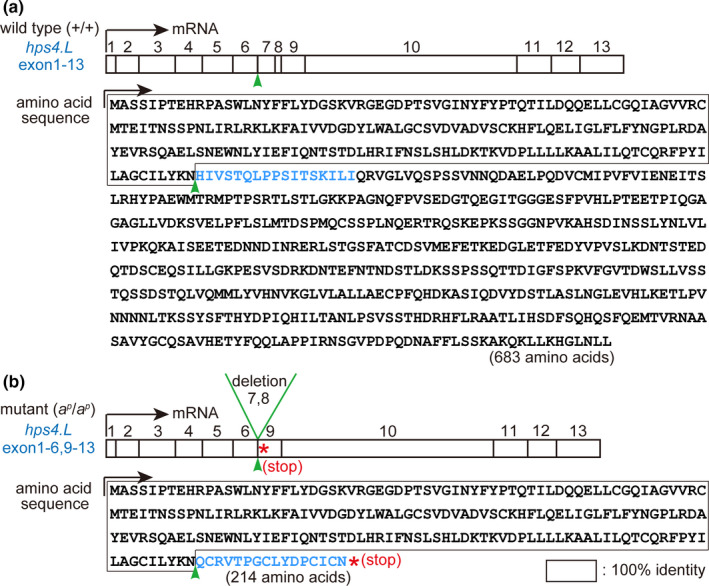
Deletion of two exons in the *hps4.L* gene in the periodic albino mutant leads to a truncated Hps4 protein. Transcripts and inferred amino acid sequences of Hps4.L in the wild type (a) and the mutant (b) are shown. In the wild type, 13 exons of *hps4.L* gene are transcribed and translated. In contrast, deletion of exons 7 and 8 (b, arrowhead) in *hps4.L* creates a premature stop codon (b, asterisk) in the mutant, resulting in a truncated Hps4 protein. Mutant Hps4 protein consists of 214 amino acids (b), which is much shorter than the wild‐type Hps4 protein consisting of 683 amino acids (a). Accession numbers: +/+ *hps4.L*, LC577762 (DDBJ); *a^p^/a^p^ hps4.L*, LC577763 (DDBJ)

The *hps4.L* gene consists of 13 exons interrupted by 12 introns in the wild‐type *X. laevis* (Figure 2c). In the present study, genomic sequencing of the *hps4.L* gene between exons 6 and 9 was carried out and compared between the wild type and the mutant. In the periodic albino mutant, a 1,920 bp deletion was detected between introns 6 and 8 of the *hps4.L* gene (Figure S1), as illustrated in Figure 2c. Mutant *hps4.L* mRNA (1,957 bp) is shorter than the wild‐type mRNA (2,052 bp), because of deletion of exons 7 and 8 in the *hps4.L* gene (Figure S2). In the mutant *hps4.L* transcript, a premature stop codon is created in exon 9 (Figure S2), resulting in a truncated Hps4 protein with a length of 214 amino acids, which is much shorter than the wild‐type protein (683 amino acids; Figure 3).

Two genes, *HPS1* and *HPS4* encode components of the biogenesis of lysosome‐related organelle complex‐3 (BLOC‐3) (Chiang et al., 2003; Martina et al., 2003; Nazarian et al., 2003). In humans, frameshift mutations or non‐sense mutations in the *HPS4* gene are known to produce truncated proteins that disrupt the function of HPS4 protein and BLOC‐3 (Anderson et al., 2003; Bachli et al., 2004; Carmona‐Rivera et al., 2011; Suzuki et al., 2002; Wu et al., 2019). It has been reported that BLOC‐3 functions as a guanine nucleotide exchange factor for Rab GTPases (Rab32 and Rab38) and that silencing of the BLOC‐3 subunits HPS1 and HPS4 results in reduced pigmentation (Gerondopoulos et al., 2012). In addition, BLOC‐3 has been indicated to be a Rab9 effector, which is involved in melanization (Kloer et al., 2010). It is suggested that the N‐terminal and C‐terminal regions of the HPS4 protein may be important for the formation of BLOC‐3 (Carmona‐Rivera et al., 2013; Kloer et al., 2010). The importance of the HPS4 C‐terminal domain in protein function is indicated by the fact that frameshift mutations causing the loss of 5 and 7 residues from the C‐terminus result in the disease phenotype of HPS4 in humans (Bachli et al., 2004; Kloer et al., 2010). Interestingly, sequences of both the N‐terminal and C‐terminal regions of HPS4 protein are conserved among mouse, human and wild‐type *X. laevis* (Figure S3). When compared with the wild‐type Hps4 protein, the mutant Hps4 protein lacks 469 amino acids, including the C‐terminal region of the wild‐type protein (Figure S3), which is reported to contain critical residues responsible for Rab9 binding in HPS4 in mice (Ohishi et al., 2019). It is reasonable to presume that the truncated Hps4 protein in periodic albino mutants may affect the function of BLOC‐3, resulting in reduced melanization.

### 2.4 Injection of wild‐type *hps4.L* mRNA into mutant embryos rescued the albino phenotype

**FIGURE 4:**
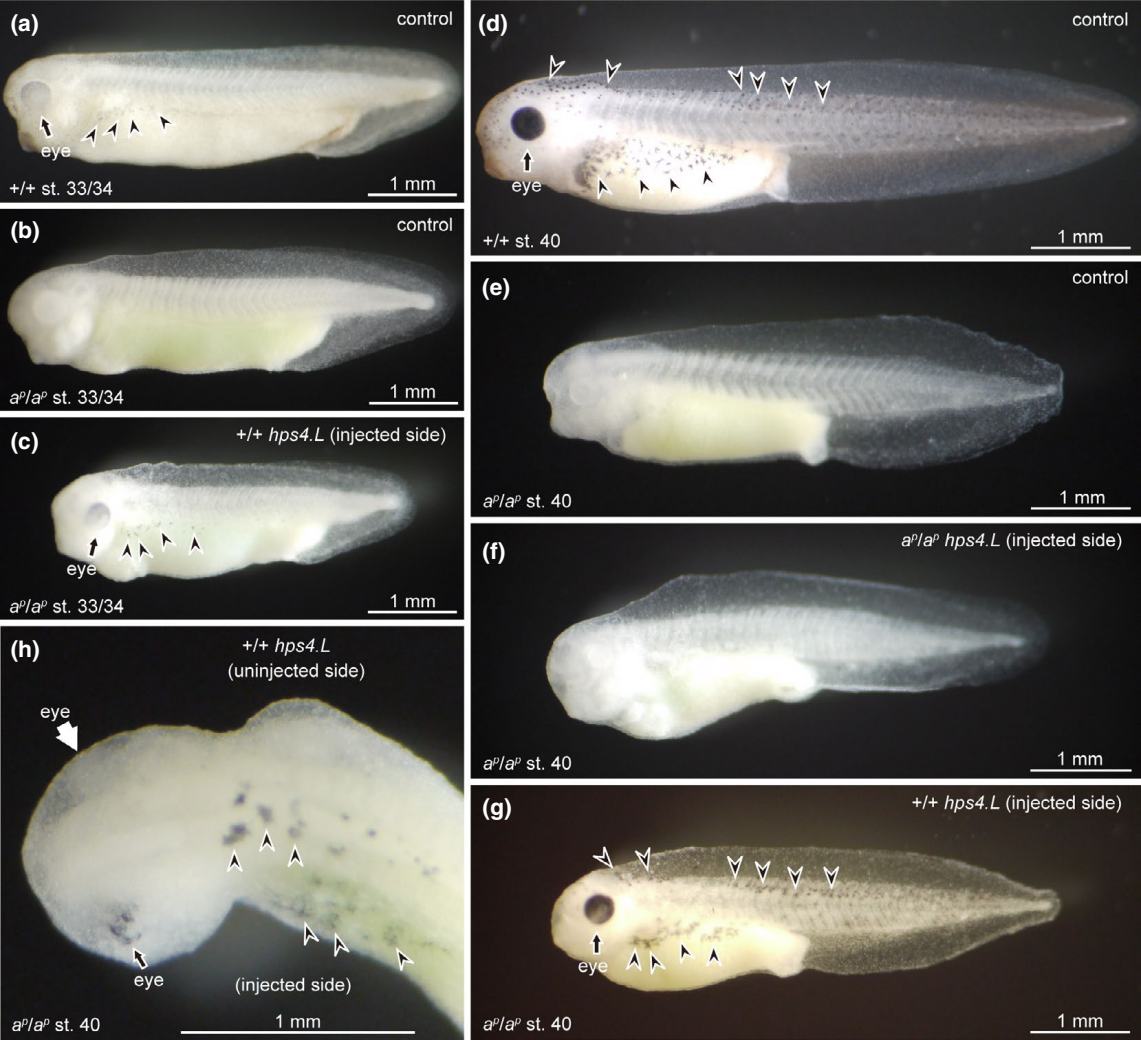
Injection of wild‐type *hps4.L* mRNA, but not mutant *hps4.L* mRNA, into mutant embryos rescued the albino phenotype. (a–c) Stage 33/34 embryos; (d–h) stage 40 embryos. Uninjected control embryos of the wild type (a, d) and the mutant (b, e) were compared with mutant embryos injected with either mutant *hps4.L* mRNA (f) or wild‐type *hps4.L* mRNA (c, g, h). Although melanin did not appear in mutant control embryos at stages 33/34 (b) and 40 (e), mutant embryos injected with wild‐type *hps4.L* mRNA expressed melanin in the eyes (black arrows) and melanophores (arrowheads) at stages 33/34 (c) and 40 (g, h). The expression of melanin in mutant embryos injected with wild‐type *hps4.L* mRNA (c, g) was similar to that of wild‐type controls (a, d). Embryos injected with mutant *hps4.L* mRNA did not express melanin at stage 40 (f), and were similar to mutant controls (e). Dorsal view of the head and trunk regions of mutant embryo injected with wild‐type *hps4.L* mRNA shows that melanin appeared in the eye of the injected area (h, black arrow), but not in the eye of the uninjected area (h, white arrow)

Rescue experiments using wild‐type *hps4.L* mRNA were carried out to determine whether *hps4.L* is a causative gene for periodic albinism. In the present study, one of the two blastomeres in two‐cell stage mutant embryos was injected with either wild‐type *hps4.L* mRNA or mutant *hps4.L* mRNA. At stage 33/34, when no melanin was present in mutant control embryos (Figure 4b), melanin appeared in the eyes and melanophores of mutant embryos injected with wild‐type *hps4.L* mRNA (Figure 4c), whose melanization was similar to wild‐type controls (Figure 4a). The time of melanin appearance in mutant embryos injected with wild‐type *hps4.L* mRNA was the same as that in wild‐type controls. At stage 40, when melanin was not present in mutant control embryos (Figure 4e), the eyes became darkly pigmented and dendritic melanophores were distributed from the head to tail in mutant embryos injected with wild‐type *hps4.L* mRNA (Figure 4g) as well as in wild‐type controls (Figure 4d). Mutant embryos injected with mutant *hps4.L* mRNA did not express melanin at stage 40 (Figure 4f) and were similar to mutant controls (Figure 4e). In mutant embryos injected with wild‐type *hps4.L* mRNA, melanin appeared in the injected area, but not in the uninjected area at stage 40 (Figure 4h); however, a few melanin‐containing cells were observed in the uninjected area, probably because these cells may have migrated from the injected area. The effect of wild‐type *hps4.L* mRNA in the rescue experiment was evident, since the eye of the injected area was darkly melanized, whereas melanin was absent in the eye of the uninjected area at stage 40 (Figure 4h). It is noteworthy that melanophores in mutant embryos injected with wild‐type *hps4.L* mRNA were heavily melanized and dendritic (Figure 4g,h) like wild‐type melanophores (Figure 4d), and were clearly different from intact mutant melanophores, which were punctate and pale in color (Figure 1h). Electron microscopic observation demonstrated that many mature melanosomes were present in melanophores of mutant embryos injected with wild‐type *hps4.L* mRNA and in wild‐type control melanophores, whereas mutant control melanophores contained premelanosomes and immature melanosomes, instead of mature melanosomes at stage 41 (Figure S4).

These findings indicate that the *hps4* gene is responsible for the periodic albino mutation of *X. laevis*. Recently, *hps4* was suggested to be a causative gene in albino catfish (Li et al., 2017). In addition, mutations of *hps5* and *hps6* have been reported to affect pigmentation in zebrafish (Daly et al., 2013) and *Xenopus tropicalis* (Nakayama et al., 2017). It is conceivable that *hps* mutations, which cause albinism, occur in poikilotherms and in mammals.

The characteristic feature of periodic albino melanophores containing many premelanosomes instead of fully melanized melanosomes may be explained by the dysfunction of the Hps1‐Hps4 complex BLOC‐3, which is required for melanosome formation from premelanosomes (Gerondopoulos et al., 2012). However, it is not clear whether Hps4 dysfunction is involved in the depigmentation of both RPE and melanophores (Hoperskaya, 1975), and in the appearance of white pigment cells, which arise from melanophore precursors in the periodic albino mutant (Fukuzawa, 2004, 2010, 2015). These questions remain to be answered.

Although mammals have only one kind of pigment cell (melanocyte), three types of pigment cells (melanophores, iridophores and xanthophores) are present in *X. laevis*. A wide variety of pigment cells is thought to originate from stem cells that contain a primordial organelle (Bagnara et al., 1979). The idea that specific pigment‐containing organelles (melanosomes, reflecting platelets and pterinosomes) may originate from a common primordial organelle is supported by the fact that mosaic pigment cells and mosaic pigment organelles are observed in many species of poikilotherms (Bagnara, 1998). Although the mechanism of melanosome biogenesis has been intensively studied in mammals (Marks et al., 2013; Raposo & Marks, 2007), processes of both reflecting platelet formation in iridophores and pterinosome formation in xanthophores in nonmammalian vertebrates are not well understood. Mutations of *HPS* are known to affect both melanosomal and nonmelanosomal LROs in mammals (Wei & Li, 2012). Therefore, it is possible that Hps4 dysfunction may be involved in abnormal pigment organellogenesis observed in melanophores, iridophores and xanthophores in the periodic albino mutation (Fukuzawa, 2006; Fukuzawa & Ide, 1986; Hoperskaya, 1981; MacMillan, 1979; MacMillan & Gordon, 1981; Seldenrijk et al., 1982). It is interesting to note that the xanthophores of the periodic albino mutant have extremely large pterinosomes containing amorphous materials or concentric lamellar structures (Fukuzawa, 2006). This is reminiscent of enlarged lamellar bodies in type II alveolar lung epithelial cells observed in *HPS1*, *HPS4* or *Rab38* mutations in mammals (Bachli et al., 2004; Guttentag et al., 2005; Osanai, 2018). In the present rescue experiment, where embryos injected with *hps4.L* mRNA were grown until stage 41, iridophores and xanthophores were not observed because these pigment cells appear later during larval development. Further studies are necessary to understand the effect of Hps4 on nonmelanosomal LROs.

Many researchers have used the periodic albino mutant for experiments such as reciprocal grafting and in situ hybridization because the absence of melanin is a useful cell marker and is suitable for observation. Reassessment of results obtained by researchers using the periodic albino may be necessary from the perspective of LRO biogenesis. Since reflecting platelets and pterinosomes are thought to be unique LROs in nonmammalian vertebrates, the periodic albino mutant may be useful for studying LRO biogenesis.

## 3 EXPERIMENTAL PROCEDURES

Wild‐type (+/+) and periodic albino mutant (*a^p^/a^p^*) *X. laevis* were purchased from Watanabe Zoshoku. *Xenopus* eggs were obtained by gonadotropin stimulation. The developmental stages were determined according to Nieuwkoop and Faber (1967).

### 3.1 Sequence analysis

*Xenopus* genes were identified using the *X. laevis* genome v9.2 (Xenbase) database. The names and symbols of the genes were based on the nomenclature guidelines described in Xenbase (http://www.xenbase.org/gene/static/geneNomenclature.jsp). Multiple sequence alignment was carried out for DNA sequences of the *hps4.L* region in chromosome 1L and the corresponding position in chromosome 1S, as well as for the amino acid sequences of mouse, human and *X. laevis* HPS4 using ClustalW multiple alignment programs provided by DDBJ (https://www.ddbj.nig.ac.jp/).

### 3.2 Isolation of *hps4.L* mRNA

Total RNA was extracted from the tails of wild‐type and mutant tadpoles (stage 47) and purified using the ISOGEN reagent with spin columns (Nippon Gene, Tokyo, Japan). Poly(A)^+^ mRNA was isolated using the Oligotex‐dT30 Super mRNA Purification Kit (Takara Bio). Double‐stranded cDNA was synthesized using the PrimeScript Double Strand cDNA Synthesis Kit (Takara Bio).

To isolate *hps4.L* transcripts from the wild type and the periodic albino mutant, polymerase chain reaction (PCR) was carried out using the following primers: 5′‐ATGGCATCCTCTATTCCTACTG‐3′ and 5′‐TTACAGCAGGTTAAGACCATG‐3′. The conditions for PCR were 94°C for 2 min 20 s, 40 cycles of 94°C for 50 s, 51°C for 50 s and 72°C for 2 min 15 s, followed by 72°C for 5 min. PCR products were subcloned into the pGEM‐T Easy Vector (Promega) and sequenced using the ABI 3730xl sequencer (Applied Biosystems).

Wild‐type *hps4.L* (DDBJ Accession Number: LC577762) and mutant *hps4.L* (DDBJ Accession Number: LC577763) cDNA fragments were used to prepare mRNAs for embryo microinjection. Synthetic capped *hps4.L* mRNAs were generated using the MEGAscript SP6 Transcription Kit (Ambion).

### 3.3 DNA sequencing of the *hps4.L* gene region between exons 6 and 9

Genomic DNA was extracted and purified from wild type and mutant embryos (stage 26/27) using the Nucleospin Tissue Kit (Macherey‐Nagel). To obtain the *hps4.L* gene between exons 6 and 9, PCR was carried out using the following primers: 5′‐CCTAGCTGGCTGCATTCTCTACA‐3′ and 5′‐GACATCCTGGGGTAACTCTGCAT‐3′. The conditions for PCR were 94°C for 2 min 20 s, 40 cycles of 94°C for 50 s, 55°C for 50 s and 72°C for 2 min 35 s, followed by 72°C for 5 min. PCR products were cloned into the pGEM‐T Easy Vector (Promega) and sequenced using the ABI 3730xl sequencer (Applied Biosystems).

### 3.4 Embryo microinjection

Embryo microinjection was carried out using the same method as previously described (Fukuzawa, 2000), with the exception that two‐cell stage mutant embryos (*a^p^/a^p^*) were used for injection of *hps4.L* mRNA. At the two‐cell stage, one of the two blastomeres was injected with either wild‐type *hps4.L* mRNA or mutant *hps4.L* mRNA using Leitz MicroManipulator (Leitz). Injected mutant embryos, as well as wild‐type and mutant control (uninjected) embryos, were grown until stage 41. Expression of melanin in the eyes and melanophores was compared between uninjected control embryos and embryos injected with wild‐type or mutant *hps4.L* mRNA.

### 3.5 Electron microscopy

Wild‐type control melanophores, mutant control melanophores and melanophores of mutant embryos injected with wild‐type *hps4.L* mRNA were examined by electron microscopy. Embryos at stage 41 were fixed in 2.5% glutaraldehyde in 0.1 M cacodylate buffer (pH 7.2) for 60 min at 4°C, post‐fixed in 2% O_S_O_4_ in the same buffer for 60 min at 4°C, dehydrated through a graded series of ethanol and embedded in epoxy resin. Ultrathin sections were stained with uranyl acetate and lead citrate, and observed using the JEOL JEM‐1010 electron microscope.

## Supporting information

Fig S1

Fig S2

Fig S3

Fig S4
