# Supplementary figures and images for: Periodic albinism of a widely used albino mutant of Xenopus laevis caused by deletion of two exons in the Hermansky–Pudlak syndrome type 4 gene
Source: Genes Cells. 2020 Nov 28;26(1):31–9. doi: 10.1111/gtc.12818 (PMC7839477; doi:10.1111/gtc.12818)

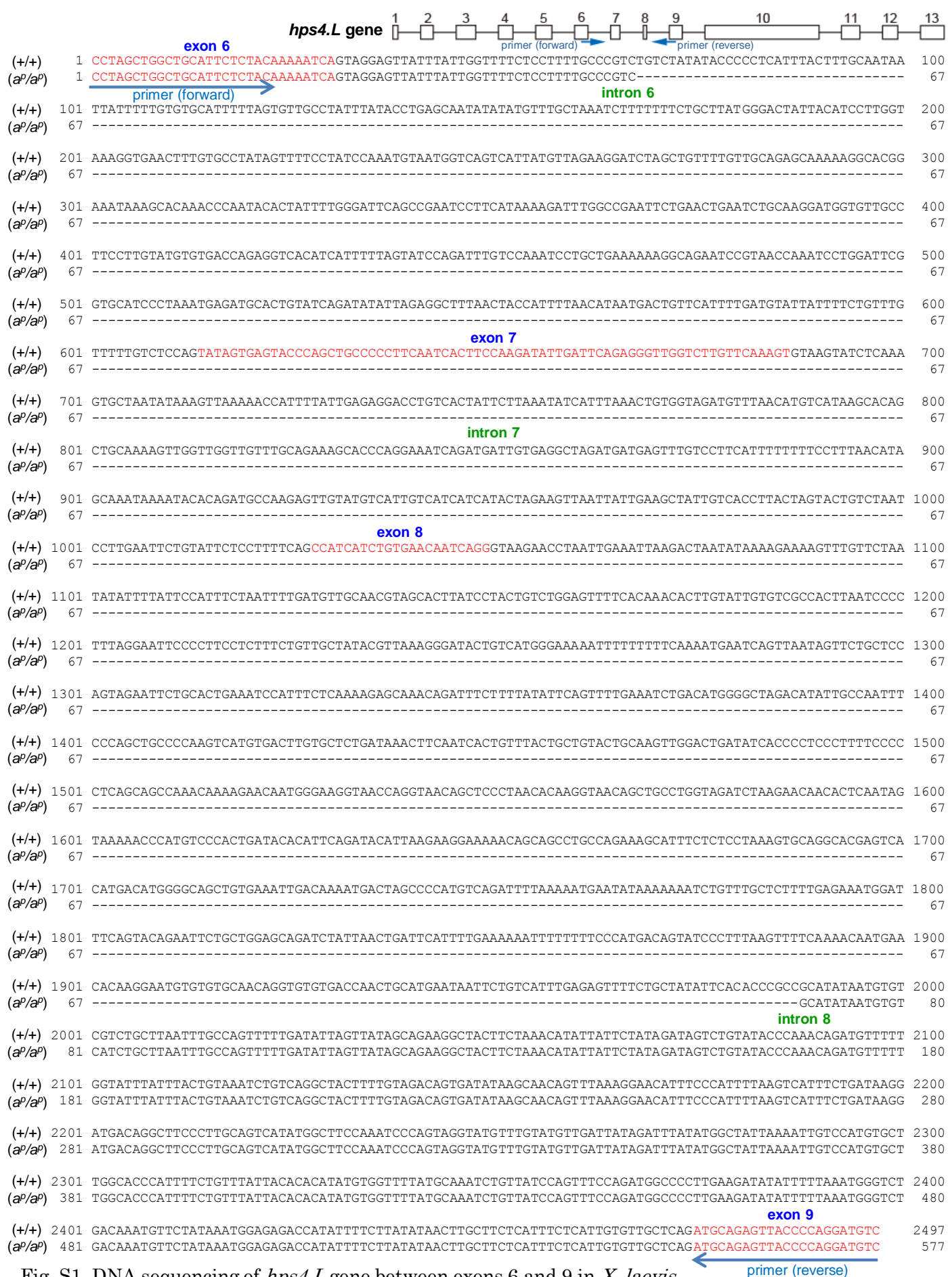

Fig. S1. DNA sequencing of *hps4.L* gene between exons 6 and 9 in *X. laevis*.

Supplement: Supplementary file 1 — Fig S1 [file GTC-26-31-s001.pdf]

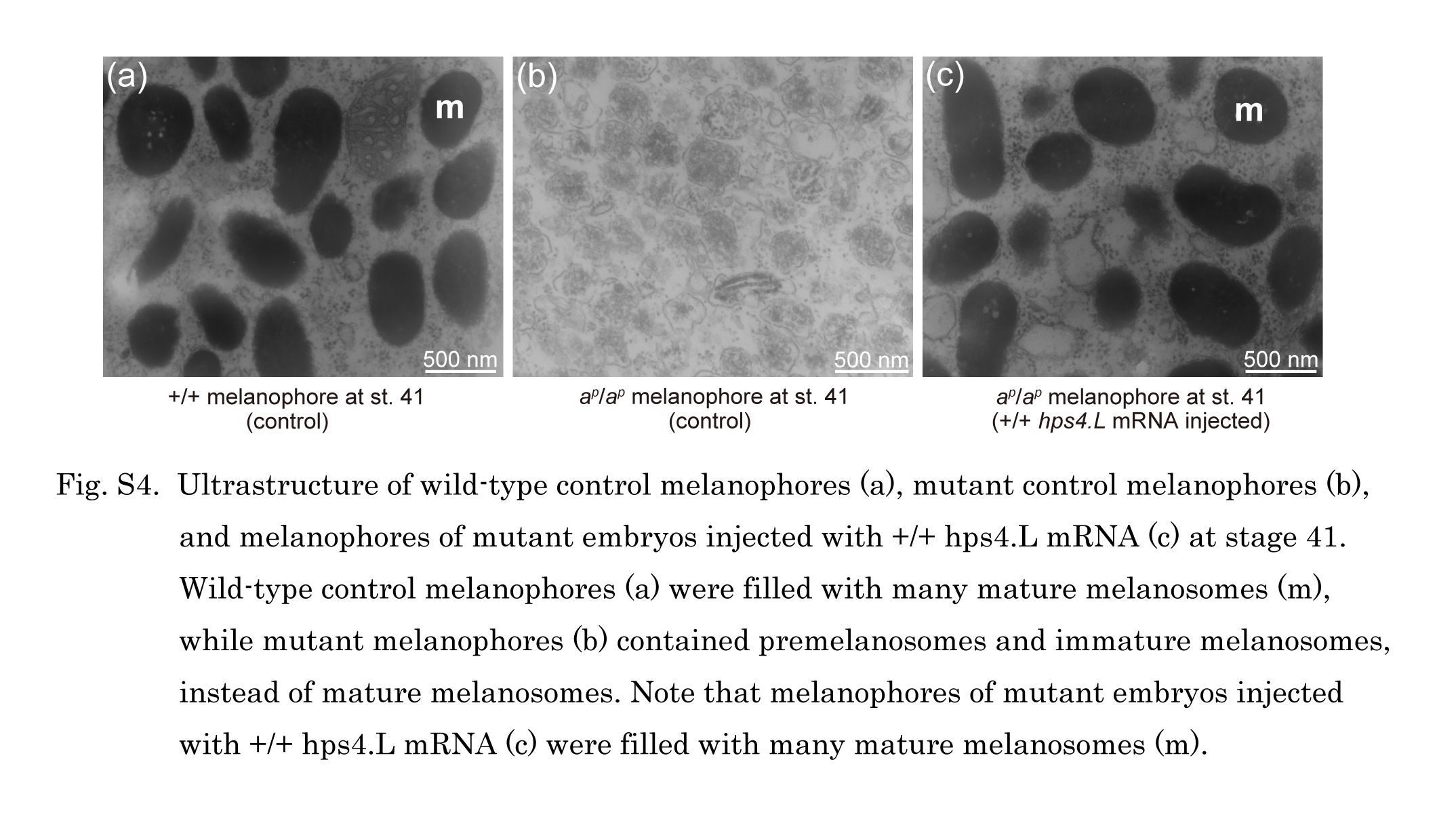

Supplement: Supplementary file 4 — Fig S4 [file GTC-26-31-s004.tif]
