# Supplementary material for: Periodic albinism of a widely used albino mutant of Xenopus laevis caused by deletion of two exons in the Hermansky–Pudlak syndrome type 4 gene
Source: Genes Cells. 2020 Nov 28;26(1):31–9. doi: 10.1111/gtc.12818 (PMC7839477; doi:10.1111/gtc.12818)
Supplement: Supplementary file 2 — Fig S2 [file GTC-26-31-s002.pdf]

hps4.L transcripts

```
          mRNA           exon 1                                   exon 2
(+/+)        1 ATGGCATCCTCTATTCCTACTGAACACAGGCCAGCATCATGGTTGAATTATTTCTTTCTTTACGATGGATCAAAGGTGAGAGGTGAAGGTGATCCCACCA  100
(ap/ap)      1 ATGGCATCCTCTATTCCTACTGAACACAGGCCAGCATCATGGTTGAATTATTTCTTTCTTTACGATGGATCAAAGGTGAGAGGTGAAGGTGATCCCACCA  100
                                                                  exon 3
(+/+)      101 GTGTTGGTATAAATTACTTCTACCCCACACAGACAATCCTTGACCAGCAGGAATTGCTGTGTGGTCAAATTGCAGGTGTTGTCCGTTGTATGACTGAGAT  200
(ap/ap)    101 GTGTTGGTATAAATTACTTCTACCCCACACAGACAATCCTTGACCAGCAGGAATTGCTGTGTGGTCAAATTGCAGGTGTTGTCCGTTGTATGACTGAGAT  200
                                                                                      exon 4
(+/+)      201 CACCAACTCTTCTCCCAACCTTATACGACTACGCAAACTGAAATTTGCCATTGTCGTTGATGGGGACTATTTGTGGGCTCTTGGGTGTTCAGTAGATGTT  300
(ap/ap)    201 CACCAACTCTTCTCCCAACCTTATACGACTACGCAAACTGAAATTTGCCATTGTCGTTGATGGGGACTATTTGTGGGCTCTTGGGTGTTCAGTAGATGTT  300
                                                                                               exon 5
(+/+)      301 GCAGATGTCAGTTGCAAGCACTTCTTACAAGAACTAATTGGGCTGTTCCTGTTCTATAATGGACCTTTGCGGGATGCCTATGAGGTCCGTTCACAGGCTG  400
(ap/ap)    301 GCAGATGTCAGTTGCAAGCACTTCTTACAAGAACTAATTGGACTGTTCCTGTTCTATAATGGACCTTTGCGGGATGCCTATGAGGTCCGTTCACAGGCTG  400

(+/+)      401 AGCTAAGCAACGAATGGAATCTTTACATTGAATTCATTCAGAACACCAGCACCGACCTACACAGGATCTTCAATTCCCTGAGTCATCTTGATAAAACTAA  500
(ap/ap)    401 AGCTAAGCAACGAATGGAATCTTTACATTGAATTCATTCAGAACACCAGCACCGACCTACACAGGATCTTCAATTCCCTGAGTCATCTTGATAAAACTAA  500
                                          exon 6
(+/+)      501 AGTAGACCCTCTCCTATTGCTGAAAGCTGCCCTCATTTTGCAGACCTGCCAGAGATTTCCTTACATCCTAGCTGGCTGCATTCTCTACAAAAATCATATA  600
(ap/ap)    501 AGTAGACCCTCTCCTATTGCTGAAAGCTGCTCTCATTTTGCAGACCTGTCAGAGATTTCCTTACATCCTAGCTGGCTGCATTCTCTACAAAAATCA----  596
                                          exon 7                                          exon 8
(+/+)      601 GTGAGTACCCAGCTGCCCCCTTCAATCACTTCCAAGATATTGATTCAGAGGGTTGGTCTTGTTCAAAGTCCATCATCTGTGAACAATCAGGATGCAGAGT  700
(ap/ap)    596 ---------------------------------------------------------------------------------------------ATGCAGAGT  605
                                          exon 9                                                    exon 10
(+/+)      701 TACCCCAGGATGTCTGTATGATCCCTGTATTTGTAATTGAAAATGAAATAACTTCACTTCGCCACTACCCTGCCGAGTGGATGACAAGAATGCCCACACC  800
(ap/ap)    606 TACCCCAGGATGTCTGTATGATCCCTGTATTTGTAATTGAAAATGAAATAACTTCACTTCGCCACTACCCTGCCGAGTGGATGACAAGAATGCCCACACC  705
                                           (stop)
(+/+)      801 TTCAAGAACATTGTCAACGCTTGGCAAAAAACCTGCAGGAAACCAGTTTCCAGTCTCAGAGGATGGTACTCAGGAAGGAATAACTGGAGGCGGGGAATCT  900
(ap/ap)    706 TTCAAGAACATTGTCAACGCTTGGCAAAAAAGCTGCAGGAAACCAGTTCCCAGTCTCAGAGGATGGTACTCAGGAAGGAATAACTGGAGGCGGGGAATCT  805

(+/+)      901 TTTCCAGTGCACCTTCCAACAGAAGAGACTCCAATACAAGGAGCAGGTGCAGGTTTACTCGTTGACAAAAGTGTAGAATTGCCCTTCCTAAGCTTAATGA 1000
(ap/ap)    806 TTTCCAGTGCACCTTCCAAAAGAAGAGACTCCAATACATGGAGCAGATGCAGGTTTACTCGTTGACAAGAGTGTAGAATTGCCCTTCCTAAGCTTAATGA  905

(+/+)     1001 CAGACAGCCCAATGCAATGTTCATCACCATTAAACCAGGAAAGGACCAGGCAATCCAAGGAACCAAAATCATCTGGTGGTAACCCAGTAAAGGCACACAG 1100
(ap/ap)    906 CAGACAGCCCAATGCAATGTTCATCACCATTAAACCAGGAAAGGACCAGGCAGTCCAAGGAACCAAAATCATCTGGTGGTAACCCAGTAAAAGCACACAG 1005

(+/+)     1101 TGATATCAACTCTTCCCTGTATAATTTGGTCCTAATTGTTCCGAAGCAGAAGGCTATATCAGAAGAGACAGAGGATAATAACGACATTAACCGAGAGCGT 1200
(ap/ap)   1006 TGATATCAACTCTTCCCTGTATAATTTGGTCCTAATTGTTCCGAAGCAGAAGGCTATATCAGAAGAGACAGAGGATAATAACGACATTAACCGAGAGCGT 1105

(+/+)     1201 TTATCTACCGGTAGCTTTGCAACGTGTGATTCTGTGATGGAATTTGAGACTAAAGAGGATGGACTGGAAACATTTGAGGACTATGTTCCAGTGTCTCTGA 1300
(ap/ap)   1106 TTATCTACCGGTAGCTTTGCAACGTGTGATTCTGTGATGGAATTTGAGACTAAAGAGGATGGACTGGAAACATTTGAGGACTATGTTCCAGTGTCTCTGA 1205

(+/+)     1301 AAGATAATACCAGTACTGAAGACCAAACAGATAGTTGTGAACAGTCCATACTGTTAGGGAAGCCTGAAAGTGTCAGTGACAGAAAAGACAACACAGAATT 1400
(ap/ap)   1206 AAGATAATACCAGTACTGAAGACCAAACAGATAGTTGTGAACAGTCCATACTGTTAGGGAAGCCTGAAAGTGTCAGTGACCGAAAAGACAACACAGAATT 1305

(+/+)     1401 TAATACCAACGACAGCACCTTAGACAAAAGTTCACCATCAAGTCAAACAACAGATATCGGTTTTTCACCAAAGGTTTTTGGGGTGACAGACTGGTCATTA 1500
(ap/ap)   1306 TAATACCAACGACAGCATCTTAGACAAAAGTTCACCATCAAGTCAAACAACAGATATCGGTTTTTCACCAAAGGTTTTTGGGGTGACAGACTGGTCATTA 1405

(+/+)     1501 CTTGTCAGCTCAACCCAGTCATCTGACAGCACACAACTGGTACAGATGATGTTATATGTCCATAATGTTAAAGGCTTAGTGTTGGCTCTCTTGGCAGAAT 1600
(ap/ap)   1406 CTTGTCAGCTCAACCCAGTCATCTGACAGCACACAACTGGTACAGATGATGTTATATGTCCATAATGTTAAAGGCTTAGTGTTGGCTCTCTTGGCAGAAT 1505
                                                                            exon 11
(+/+)     1601 GTCCTTTCCAACATGACAAGGCCTCAATTCAGGATGTGTATGACAGCACCTTGGCTTCTCTCAATGGCCTAGAAGTTCATCTTAAAGAGACTTTGCCAGT 1700
(ap/ap)   1506 GTCCTTTCCAACATGACAAGGCCTCAATTCAGGATGTGTATGACAGCACCTTGGCTTCTCTCAATGGCCTAGAAGTTCATCTTAAAGAGACTTTGCCAGT 1605
                                                                                                 exon 12
(+/+)     1701 CAACAACAATAATCTTACAAAGAGCAGCTACAGCTTTACCCATTATGATCCTATTCAGCATATTCTCACTGCCAACCTCCCGTCTGTTTCAAGCACGCAT 1800
(ap/ap)   1606 CAACAACAATAATCTTACAAAGAGCAGCTACAGCTTTACCCATTATGATCCTATTCAGCATATTCTCACTGCCAACCTCCCGTCTGTTTCAAGCACGCAT 1705
                                                                                                 exon 13
(+/+)     1801 GACCGCCACTTCCTCCGAGCTGCCACTCTGATACATTCCGACTTTAGTCAACATCAGTCTTTCCAGGAAATGACAGTAAGAAATGCAGCCTCTGCCGTCT 1900
(ap/ap)   1706 GACCGCCACTTCCTCCGAGCTGCCACTCTGATACATTCCGACTTTAGTCAACATCAGTCTTTCCAGGAAATGACAGTAAGAAATGCAGCCTCTGCCGTCT 1805

(+/+)     1901 ATGGCTGCCAAAGTGCTGTCCATGAGACCTACTTCCAGCAGCTGGCACCACCTATAAGGAACTCTGGAGTCCCTGACCCACAGGACAATGCATTCTTCTT 2000
(ap/ap)   1806 ATGGCTGCCAAAGTGCTGTCCATGAGACCTACTTCCAGCAGCTGGCACCACCTATAAGGAACTCTGGAGTCCCTGACCCACAGGACAATGTATTCTTCTT 1905

(+/+)     2001 ATCCAGCAAGGCAAAGCAAAAACTACTGAAACATGGTCTTAACCTGCTGTAA(stop)                                              2052
(ap/ap)   1906 ATCCAGCAAGGCAAAGCAAAAACTACTGAAACATGGTCTTAACCTGCTGTAA                                                    1957
```

Fig. S2. Comparison of *hps4.L* transcripts between wild type (+/+) and mutant (a^p/a^p) *X. laevis.* In the mutant *hps4.L* transcript lacking exons 7 and 8, a premature stop codon is created in exon 9. Accession numbers: +/+ *hps4.L,* LC577762 (DDBJ); a^p/a^p *hps4.L,* LC577763 (DDBJ)
