# Supplementary material for: Periodic albinism of a widely used albino mutant of Xenopus laevis caused by deletion of two exons in the Hermansky–Pudlak syndrome type 4 gene
Source: Genes Cells. 2020 Nov 28;26(1):31–9. doi: 10.1111/gtc.12818 (PMC7839477; doi:10.1111/gtc.12818)
Supplement: Supplementary file 3 — Fig S3 [file GTC-26-31-s003.pdf]

```
mouse             1 MATTTPPETKSAAWWNYFFLYDGSKVKGEGDPTRAGICYFYPPQTLLDQQELLCGQLAGV  60
human             1 MATSTSTEAKSASWWNYFFLYDGSKVKEEGDPTRAGICYFYPSQTLLDQQELLCGQIAGV  60
X.laevis(+/+)     1 MASSIPTEHRPASWLNYFFLYDGSKVRGEGDPTSVGINYFYPTQTILDQQELLCGQIAGV  60
X.laevis(aP/aP)   1 MASSIPTEHRPASWLNYFFLYDGSKVRGEGDPTSVGINYFYPTQTILDQQELLCGQIAGV  60

mouse            61 VRCLWDLSGTPPMLIRMRNLKFAIRADGDYLWALGCGVEISDASCRQFLDQLIGFFHFYM 120
human            61 VRCVSDISDSPPTLVRLRKLKFAIKVDGDYLWVLGCAVELPDVSCKRFLDQLVGFFNFYN 120
X.laevis(+/+)    61 VRCMTEITNSSPNLIRLRKLKFAIVVDGDYLWALGCSVDVADVSCKHFLQELIGLFLFYN 120
X.laevis(aP/aP)  61 VRCMTEITNSSPNLIRLRKLKFAIVVDGDYLWALGCSVDVADVSCKHFLQELIGLFLFYN 120

mouse           121 GPVSLAYKSHPQEELSLQWDTSITQVLRSTSESHRIFNALWNLDRTKVEPLLLLKAALIL 180
human           121 GPVSLAYENCSQEELSTEWDTFIEQILKNTSDLHKIFNSLWNLDQTKVEPLLLLKAARIL 180
X.laevis(+/+)   121 GPLRDAYEVRSQAELSNEWNLYIEFIQNTSTDLHRIFNSLSHLDKTKVDPLLLLKAALIL 180
X.laevis(aP/aP) 121 GPLRDAYEVRSQAELSNEWNLYIEFIQNTSTDLHRIFNSLSHLDKTKVDPLLLLKAALIL 180

mouse           181 QTCQRSPHVLAGCILYKGLIVNSQLLPSLTAKVLLHQTVPA-DQRLPGAGAAPQETGAAL 239
human           181 QTCQRSPHILAGCILYKGLIVSTQLPPSLTAKVLLHRTAPQ-EQRLPTGEDAPQEHGAAL 239
X.laevis(+/+)   181 QTCQRFPYILAGCILYKNHIVSTQLPPSITSKILIQRVGLVQSPSSVNNQDAELPQDV-- 238
X.laevis(aP/aP) 181 QTCQRFPYILAGCILYKNQCRVTPGCLYDPCICN                           214

mouse           240 PPDVQITSVFLSEEEVASLHEFPVEHETRL---------QGSS-VQYPP-WDQS----SP 284
human           240 PPNVQIIPVFVTKEEAISLHEFPVEQMTRSLASPA--GLQDGS-AQHHP-KGGSTSALKE 295
X.laevis(+/+)   239 --CMIPVFVIENEITSLRHYP--AEWMTRMPTPSRTLSTLGKKPAGNQFPVSEDGTQEGI 294

mouse           285 TQAEDAWASAAIPEPTPH-DGA--CPSGSGA-----DERLPRLEQECAGPTGLCTTACGQ 336
human           296 N-ATGHVESMAWTTPDPT-SPDEACPDGRKE-----NGCLSGHDLESIRPAGLHNSARGE 348
X.laevis(+/+)   295 TGGGESFPVHLPTEETPIQGAGAGLLVDKSVELPFLSLMTDSPMQCSSPLNQERTRQSKE 354

mouse           337 GSGLSSRLQKELCLSREELDSSE-----------MHVSEAQEAFPPLPALGDLETLH-S- 383
human           349 VLGLSSSLGKELVFLQEELDLSEIHIPEAQEVEMASGHFAFLHVPVP-DGRAPYCKA-SL 406
X.laevis(+/+)   355 PKSSGGNPVK-----------------------AHSDIN--SSLYNLVLIVPKQKAISE 388

mouse           384 ---SHSAPTLPEDTAIC-SCLHPCPLERLPESGRLGQLADLPLTNGQTQVPGTDPLPSSM 439
human           407 SASSSLEPTPPEDTAIS-SLRPPSAPEMLTQHGAQEQLEDHPGHSSQAPIPRADPLPRRT 465
X.laevis(+/+)   389 ETEDNNDIN---RERLSTGSFATCDSVMEFETKEDGLE--TFEDYVPVSLKDNTSTEDQT 443

mouse           440 PVALPPQHPVGVEPSVEP---------YGNGAQE-SHSALPR--SSRSPDSPGPSPS--- 484
human           466 RRPLLLPRLDPGQRGNKLPTGEQGLDEDVDGVCE-SHAAPGLECSSGSANCQGAGPS--- 521
X.laevis(+/+)   444 DSCEQSILLGKPESVS-----------DRKDNTEFNTND--STLDKSSPSSQTTDIGFSP 490

mouse           485 ----ADRTG--FKPSPSGRHAGLVPMNLYTHSVNGLVLSLLAEETLLSDTAAIEEVYHSS 538
human           522 ----ADGIS--SRLTPAESCMGLVRMNLYTHCVKGLVLSLLAEEPLLGDSAAIEEVYHSS 575
X.laevis(+/+)   491 KVFGVTDWSLLVSSTQSSDSTQLVQMMLYVHNVKGLVLALLAECPFQHDKASIQDVYDST 550
                      *  *
mouse           539 LASLNGLEVHLKETLPRDEASLTSSTYNFLHYDRIQSVL-----SANLPLVTAPQDRRFL 593
human           576 LASLNGLEVHLKETLPRDEAASTSSTYNFTHYDRIQSLL-----MANLPQVATPQDRRFL 630
X.laevis(+/+)   551 LASLNGLEVHLKETLPVNNNNLTKSSYSFTHYDPIQHILTANLPSVSSTHDRHFLRAATL 610

mouse           594 QAVNLMHSDFALLPMLYEMTIRNASTAVYACSSPAQETYFQQLAPTARSSGFPNPQDCAF 653
human           631 QAVSLMHSEFAQLPALYEMTVRNASTAVYACCNPIQETYFQQLAPAARSSGFPNPQDGAF 690
X.laevis(+/+)   611 IHSDFSQHQ-----SFQEMTVRNAASAVYGCQSAVHETYFQQLAPPIRNSGVPDPQDNAF 665

mouse           654 SLAGKAKQKLLKHGVNLL  671
human           691 SLSGKAKQKLLKHGVNLL  708
X.laevis(+/+)   666 FLSSKAKQKLLKHGLNLL  683
```

Fig. S3. Amino acid sequence alignment of HPS4 from mouse, human, wild-type and mutant *X. laevis*. The Hps4 protein of mutant *X. laevis* lacks amino acid residues 215–683, when compared with that of wild-type *X. laevis*. Identical residues are shaded. Asterisks (*) show critical residues reported to be responsible for Rab9 binding in HPS4 in mice (Ohishi et al., 2019). Reference sequences: mouse HPS4, NP_001346782.1; human HPS4, NP_001336825.1 Accession numbers: +/+ *X. laevis hps4.L*, LC577762 (DDBJ); a^p/a^p *X. laevis hps4.L*, LC577763 (DDBJ)
